# Lower Extremity Reconstruction

**Published:** 2013-02-18

**Authors:** Adam M. Feintisch, Ramazi Datiashvili

**Affiliations:** Department of Surgery, Division of Plastic Surgery, New Jersey Medical School, University of Medicine and Dentistry of New Jersey, Newark

## DESCRIPTION

A 39-year-old male with an open wound of the left lower extremity complicated by exposed hardware following open reduction and internal fixation of a tibial plateau fracture.

## QUESTIONS

1. **How are soft tissue injuries in relation to fractures of the lower extremity classified?**
2. **What factors must be taken into account when considering limb salvage?**
3. **When is the optimal time for soft tissue coverage following traumatic lower extremity wounds?**
4. **What are the options for soft tissue coverage in lower extremity reconstruction?**

## DISCUSSION

Treatment of high-energy trauma to the lower-extremity with associated soft-tissue and bony injury poses a significant problem. An assessment of limb viability is made with careful examination of the vascular, bone, soft-tissue, and nerve injuries. The goal in treatment of a severely injured lower extremity is to return the patient to ambulatory status. The decision of limb salvage versus amputation while maintaining maximal functional length is a critical one.^1^^,^^5^

When referring to long bone fracture pattern and soft-tissue injury, the Gustilo-Anderson fracture classification system is typically employed to help describe injuries and prognosis.^1^^,^^2^ Although a grade IIIA injury entails an open fracture with associated extensive soft-tissue damage, because there is no paucity of soft-tissue coverage, plastic surgeons are not typically consulted. Instead, assistance is largely needed when the grade is a Gustilo IIIB (III with soft-tissue loss, periosteal stripping, and bone exposure) or IIIC (III with arterial injury requiring repair).^1^ Although widely used, this classification scheme has limitations. Limitations include an inability to accurately describe injuries, delineate the necessity of arterial repair (single vs multiple-vessel injury), or take into account nerve injury.

To predict the outcome of salvage efforts for mangled extremities to help properly identify patients who would benefit from primary limb amputation versus reconstruction, and to attempt a better classification scheme, numerous scoring systems have been proposed. These include the Mangled Extremity Syndrome Index, Mangled Extremity Severity Score amongst others.^1^^,^^2^ Unfortunately, these systems are not reliable predictors of limb salvage, amputation, or functional recovery. Therefore, scoring based on these systems should not be considered prognostic, and decisions should be made on an individual basis. The main indications for salvage remain adults with soft-tissue and bony injury with preserved sensation and any injured limb in the pediatric population.^2^ Although not an absolute indication for amputation, nerve laceration remains a relative contraindication to salvage. Before determining that a limb is completely insensate, such injuries should be managed with debridement, vascular reconstruction, nerve repair (whether primary or with nerve grafts), and orthopedic fixation when applicable.^2^

After bony stabilization, vascular repair, and adequate debridement, immediate soft-tissue coverage is indicated when hardware or vital structures are exposed.^2^^,^^4^^,^^5^ If neither are exposed or the zone of injury is still uncertain, serial debridement should be performed prior to definitive coverage.^1^^,^^3^ Although some authors have failed to show an effect of reconstruction timing on ultimate outcome, the majority agree that a lower complication rate is seen with early soft-tissue coverage.^1^^,^^2^ Godina stressed that radical debridement and closure of wounds within 72 hours after injury had the lowest complication and highest success rates.^2^^,^^3^ When vital structures (ie: nerves, joints, vessels, tendons, denuded bone) or hardware are exposed, expeditious coverage remains the gold standard. Definitive timing of wound closure, especially with microsurgical reconstruction, should be determined by the general condition of the patient and the wound.^1^^,^^2^^,^^4^

Flap choice for lower extremity wound coverage is less important than wound bed quality and the flap's ability to deliver well-perfused tissue. Wounds around the knee may be coveraged by using the medial or lateral head of a gastrocnemius muscle flap. Care must be taken to avoid common peroneal nerve injury when using lateral muscle. If unavailable, vastus lateralis or anterior lateral thigh flaps using retrograde flow through the descending branch of the lateral circumflex femoral artery are other alternatives. Defects around the knee and lower thigh may also be reconstructed with a lateral genicular artery flap. Although free flaps are not typically used in this region, they remain an option when additional flaps are unavailable.^2^^,^^6^^-^8

After bony stabilization, tibial defects may be reconstructed using a variety of flaps. In general, the tibial region is divided into thirds. Defects in the proximal third are best reconstructed using a gastrocnemius muscle flap. Defects in the middle third are typically repaired using pedicled gastrocnemius or soleus muscle flaps; however, as high-energy wounds in this region typically manifest with extensive zones of injury, local flaps may not be dependable. Free flap reconstruction would then be preferred. Options include rectus abdominis, scapula, anterior lateral thigh, gracilis, and latissimus muscle flaps. These are also preferred for wounds at the distal third of the tibia because of limited local tissue availability. For those in whom free flap reconstruction is not an option, a reversed sural flap may be considered. Foot and ankle reconstruction overlaps with reconstruction in the distal third of the leg, however, debulking of free flaps is often necessary to optimize form and function.^1^^,^^2^^,^^6^^-^8

Our patient is a 39-year-old male status post fall from height resulting in bilateral calcaneal fractures and a left tibial plateau fracture. The patient underwent open reduction and internal fixation of his left tibial plateau fracture. He developed an open wound of the left lateral proximal third of his leg resulting in exposed hardware. After serial debridement, the limb was salvaged using a lateral gastrocnemius muscle flap and split-thickness skin autograft.

## Figures and Tables

**Figure 1:**
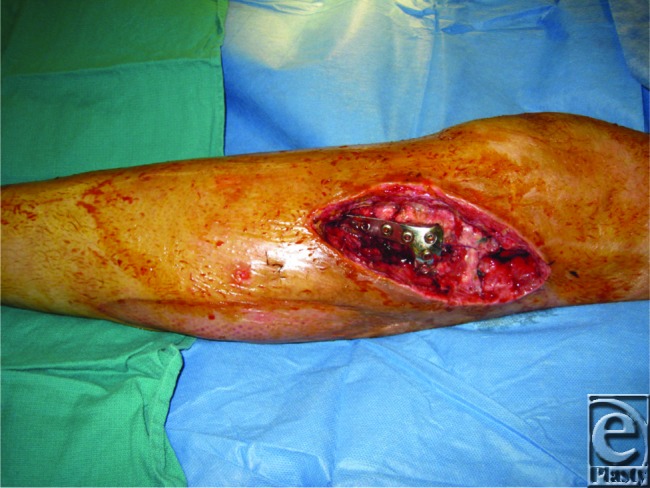
Lower extremity defect status post serial debridement. Exposed hardware can be seen.

**Figure 2:**
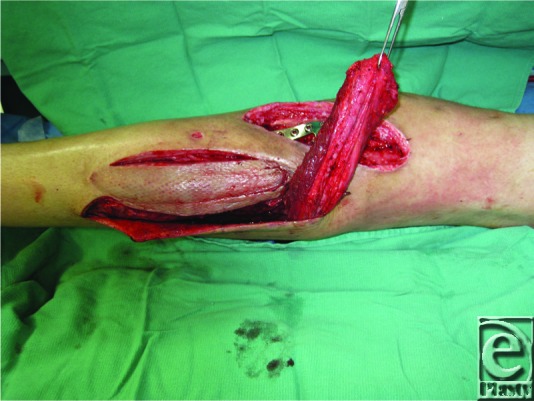
Lateral gastrocnemius muscle flap.

**Figure 3:**
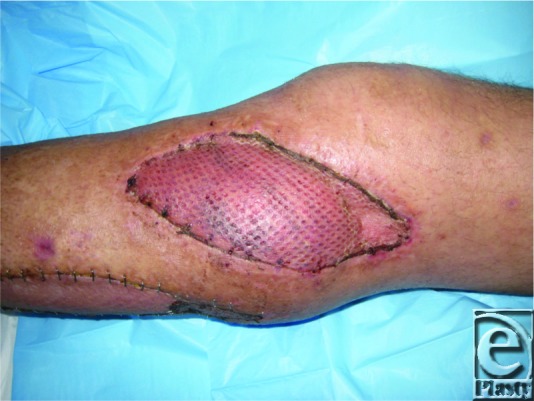
Postoperative result at 1 month showing split-thickness skin graft.

## References

[1] Kasabian AK, Karp NS, Thorne CH, Bartlett SP, Beasley RW, Aston SJ, Gurtner GC, Spear SL (2007). Lower-extremity reconstruction. Grabb and Smith's Plastic Surgery.

[2] Hollenbeck ST, Toranto JD, Taylor BJ (2011). Perineal and lower extremity reconstruction. Plast Reconsr Surg.

[3] Godina M (1986). Early microsurgical reconstruction of complex trauma of the extremities. Clin Plast Surg.

[4] Sisco M, Howard MA, Kryger ZB, Sisco M (2007). Management of exposed and infected orthopedic protheses. Practical Plastic Surgery.

[5] Sisco M, Howard MA, Kryger ZB, Sisco M (2007). Lower extremity reconstruction. Practical Plastic Surgery.

[6] Mackenzie DJ, Seyfer AE, McCarthy JG (2006). Reconstructive surgery: lower extremity coverage. Mathes Plastic Surgery.

[7] Wheeless CR (2011). Soft tissue coverage for the leg. Wheeless' Textbook of Orthopaedics.

[8] Attinger CE, Ducic I, Thorne CH, Bartlett SP, Beasley RW, Aston SJ, Gurtner GC, Spear SL (2007). Foot and ankle reconstruction. Grabb and Smith's Plastic Surgery.

